# Development and validation of prediction models for papillary thyroid cancer structural recurrence using machine learning approaches

**DOI:** 10.1186/s12885-024-12146-4

**Published:** 2024-04-08

**Authors:** Hongxi Wang, Chao Zhang, Qianrui Li, Tian Tian, Rui Huang, Jiajun Qiu, Rong Tian

**Affiliations:** 1 Department of Nuclear Medicine, West China Hospital, Sichuan University, No 37. Guoxue Alley, 610041 Chengdu, China; 2 West China Biomedical Big Data Center, West China Hospital, Sichuan University, 610041 Chengdu, China

**Keywords:** Papillary thyroid cancer, Recurrence, Machine learning, Prediction model

## Abstract

**Background:**

Although papillary thyroid cancer (PTC) patients are known to have an excellent prognosis, up to 30% of patients experience disease recurrence after initial treatment. Accurately predicting disease prognosis remains a challenge given that the predictive value of several predictors remains controversial. Thus, we investigated whether machine learning (ML) approaches based on comprehensive predictors can predict the risk of structural recurrence for PTC patients.

**Methods:**

A total of 2244 patients treated with thyroid surgery and radioiodine were included. Twenty-nine perioperative variables consisting of four dimensions (demographic characteristics and comorbidities, tumor-related variables, lymph node (LN)-related variables, and metabolic and inflammatory markers) were analyzed. We applied five ML algorithms—logistic regression (LR), support vector machine (SVM), extreme gradient boosting (XGBoost), random forest (RF), and neural network (NN)—to develop the models. The area under the receiver operating characteristic (AUC-ROC) curve, calibration curve, and variable importance were used to evaluate the models’ performance.

**Results:**

During a median follow-up of 45.5 months, 179 patients (8.0%) experienced structural recurrence. The non-stimulated thyroglobulin, LN dissection, number of LNs dissected, lymph node metastasis ratio, N stage, comorbidity of hypertension, comorbidity of diabetes, body mass index, and low-density lipoprotein were used to develop the models. All models showed a greater AUC (AUC = 0.738 to 0.767) than did the ATA risk stratification (AUC = 0.620, DeLong test: *P* < 0.01). The SVM, XGBoost, and RF model showed greater sensitivity (0.568, 0.595, 0.676), specificity (0.903, 0.857, 0.784), accuracy (0.875, 0.835, 0.775), positive predictive value (PPV) (0.344, 0.272, 0.219), negative predictive value (NPV) (0.959, 0.959, 0.964), and F1 score (0.429, 0.373, 0.331) than did the ATA risk stratification (sensitivity = 0.432, specificity = 0.770, accuracy = 0.742, PPV = 0.144, NPV = 0.938, F1 score = 0.216). The RF model had generally consistent calibration compared with the other models. The Tg and the LNR were the top 2 important variables in all the models, the N stage was the top 5 important variables in all the models.

**Conclusions:**

The RF model achieved the expected prediction performance with generally good discrimination, calibration and interpretability in this study. This study sheds light on the potential of ML approaches for improving the accuracy of risk stratification for PTC patients.

**Trial registration:**

Retrospectively registered at www.chictr.org.cn (trial registration number: ChiCTR2300075574, date of registration: 2023-09-08).

**Supplementary Information:**

The online version contains supplementary material available at 10.1186/s12885-024-12146-4.

## Background

Papillary thyroid cancer (PTC) is one of the most common types of differentiated thyroid cancer (DTC), accounting for more than 90% of DTC. Although the mortality rate for PTC patients is low, 10–30% of PTC patients will still experience recurrence or metastasis after initial treatment [1], which is the main cause of death in PTC patients. Therefore, accurate risk stratification and individualized treatment and follow-up strategies are essential for detecting recurrent disease early and improving the prognosis of PTC patients.

The 2009 American Thyroid Association (ATA) guidelines proposed a three-category system to estimate the likelihood of DTC patients developing structural recurrence during postoperative follow-up [2], and a revised ATA risk stratification system was proposed in 2015 [3]; this system has been widely used and validated in clinical practice. Although the ATA system is flexible and can easily estimate risk based only on surgical/histological findings, several prognostic factors in the system do not specify the cutoff values for risk stratification, especially considering heterogeneity among PTC patients; for example, thyroglobulin (Tg) is a specific product of thyroid follicular cells, and it has been demonstrated that a high level of postoperative thyroid stimulating hormone (TSH)-suppressed Tg is associated with a high risk of recurrent disease and mortality, with a wide range of suppressed Tg cutoff values [3]. Moreover, the prognostic value of several prognostic factors remains controversial in PTC, such as metastatic lymph node (LN) features (i.e., the LN metastasis ratio and extranodal extension) [4–6], inflammation-based markers (i.e., the neutrophil-to-lymphocyte ratio [NLR], the platelet-to-lymphocyte ratio [PLR], the lymphocyte-to-monocyte ratio [LMR], and the prognostic nutritional index [PNI]) [7–9], and metabolic-related markers (i.e., obesity and dyslipidemia) [10, 11], which therefore need to be confirmed.

In the age of precision medicine, there is considerable enthusiasm for estimating prognosis by relying on models that can simultaneously consider many factors and provide an estimate of absolute risk [12]. Machine learning (ML) provides a novel approach to achieve this goal and has advantages in incorporating a larger number of multidimensional variables with more dynamic interactions than traditional prognostic tools [13]. Briefly, ML accounts for partial, nonlinear relationships, or multiple coexistent states between variables and outcomes, and each variable in an ML model can have a variable weight according to the changes in other variables; therefore, it can realize more individual predictions [14].

ML approaches have already proven to be effective predictive tools for various types of tumors [15–17]; thus, ML models may also prove useful in PTC risk stratification. However, to the best of our knowledge, only a few studies have developed ML models for predicting death or recurrence in patients with thyroid cancer [18–23]. In addition, previous studies were partially limited by the lack of large datasets, single algorithms, inadequate variables, and incomplete model evaluation [18–23], which hindered clinicians from better understanding the application of ML in prognosis prediction for PTC. Thus, this study aimed to develop and validate multiple ML models to predict structural recurrence in PTC patients based on a large sample of PTC patients with comprehensive clinical variables.

## Methods

### Study design and population

**Fig. 1:**
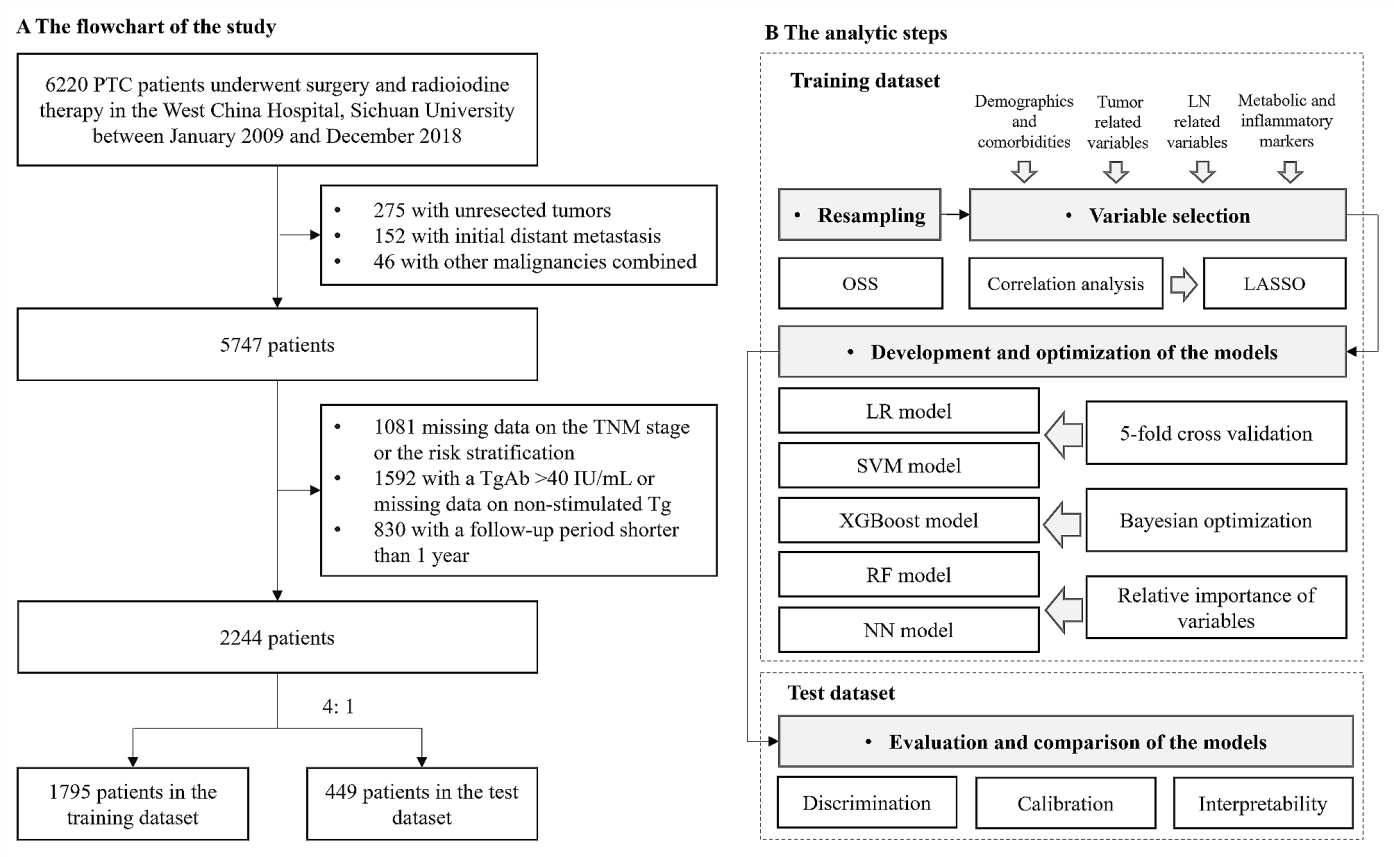
Flowchart of the study (**A**) and the analytic steps (**B**). PTC, papillary thyroid cancer; Tg, thyroglobulin; TgAb, anti-thyroglobulin antibody; LASSO, least absolute shrinkage and selection operator; LR, logistic regression; XGBoost, eXtreme gradient boosting; SVM, support vector machine; RF, random forest; NN, neural network; AUCs, area under the receiver operating characteristic curves

The electronic medical records of patients with thyroid cancer treated at West China Hospital, Sichuan University, were fully screened to retrospectively review all PTC patients treated and followed up. We restricted our analyses to PTC patients who underwent thyroid surgery (with or without lymph node dissection) and radioiodine (^131^I) therapy at West China Hospital, Sichuan University between January 2009 and December 2018 (*n* = 6220). We excluded patients with unresected tumors (*n* = 275), initial distant metastasis (*n* = 152), or other malignancies combined (*n* = 46), patients with unavailable information about the 8th edition of the AJCC TNM staging system [24] or the 2015 ATA risk stratification [3] (*n* = 1081), patients with a positive TgAb (> 40 IU/mL) [25] or missing data on postoperative non-stimulated Tg (TSH < 30 µIU/ml) (*n* = 1592), and patients with a follow-up period shorter than 1 year (*n* = 830). Finally, a total of 2244 patients were included in the prediction models (Fig. 1A).

This study was conducted following the basic principles of the Declaration of Helsinki and was approved by the West China Hospital Clinical Trials and Biomedical Ethics Committee, Sichuan University (Approval in 2020, No. 678). Written informed consent was waived given the retrospective nature of the study.

### Potential input variables

To include as many predictive variables as possible and ensure the availability and representation of variables from the database. We used 29 potential input variables consisting of four dimensions in this study: (1) demographic characteristics and comorbidities, including age, sex, race, smoking status, alcohol drinking status, comorbidity of diabetes status, comorbidity of hypertension status, and comorbidity of Hashimoto’s thyroiditis; (2) tumor-related variables, including histology, tumor diameter, tumor foci, tumor location, external thyroid invasion (ETE), BRAF (V600E) mutation, and postoperative non-stimulated Tg; (3) LN-related variables, including LN dissection, number of LNs dissected, extranodal extension (ENE), lymph node metastasis ratio (LNR), and N stage; and (4) metabolic and inflammatory markers, including body mass index (BMI), triglyceride, cholesterol, low-density lipoprotein (LDL), high-density lipoprotein (HDL), neutrophil–lymphocyte ratio (NLR), platelet–lymphocyte ratio (PLR), lymphocyte–monocyte ratio (LMR), and prognostic nutritional index (PNI).

The missing values of potential input variables are represented as a category when the missing value was ≥ 20%, and multiple imputations were used when the missing value was < 20%, as previously reported [26, 27] (Supplementary Data 1). Receiver operating characteristic (ROC) curve analysis was performed, and the Youden index was used to determine the cutoff value for continuous variables [28]. The details of the potential input variables are shown in Supplementary Data 2.

### Definition of structural recurrence

The outcome of this study was structural recurrence that occurred during follow-up after initial thyroid surgery. According to the 2015 ATA guidelines [3], patients were considered to have structural recurrent disease if any of the following conditions were met: (1) structural disease confirmed by cytology/histology; (2) highly suspicious lymph nodes or thyroid bed nodules on neck ultrasound; or (3) highly suspicious metastatic disease on whole-body ^131^I scintigraphy, ^18^fluorodeoxyglucose positron emission tomography scans, or other cross-sectional imaging. The last follow-up date of this study was July 31, 2021.

### Dataset split and resampling

Figure 1B shows the analysis steps of this study. Patients were randomly divided into a training dataset and a test dataset according to a 4 to 1 ratio. The training dataset was used to develop and optimize the models (*n* = 1795), and the test dataset was used to validate and compare the models (*n* = 449). To ensure that the models can effectively predict the outcome (minority class), one-sided selection (OSS) under-resampling method was used to establish a balanced training dataset [29] (Supplementary Data 3).

### Variable selection

Spearman correlation analysis was used to evaluate the correlation of all variables with each other. Spearman’s correlation coefficients range from -1 to 1, and values of coefficients close to -1 or 1 represent stronger relationships than values closer to zero. Then, the least absolute shrinkage and selection operator (LASSO) method was used to select the input variables. LASSO formulates curve fitting as a quadratic programming problem, where the objective function penalizes the absolute size of the regression coefficients based on the value of a tuning parameter λ involved in the maximum AUC value [30]. Thus, LASSO can perform automatic variable selection by driving the coefficients of irrelevant variables to zero.

### Development and optimization of the models

Five-fold cross-validation was used to avoid training overfitting, and the training dataset was divided into 5 equal parts, this process was repeated 5 times. In the first step, the first part was used for validation, and the remaining parts were used for training. Similarly, the second part was used for validation in the second fold, and this process was continued for the rest of the folding. Five popular ML algorithms were applied to develop models based on selected variables, including logistic regression (LR), support vector machine (SVM) [31], eXtreme gradient boosting (XGBoost) [32], random forest (RF) [33], and neural network (NN) [34]. The hyperparameters for the five models were optimized via Bayesian optimization (BO), and the models were trained on a training set for optimization and validated on a validation set for each hyperparameter configuration. The ideal parameter setup provided the highest AUC values [35] (Supplementary Data 4). Finally, the relative importance of the variables of each model was ranked, which can reflect the contribution of each variable when predicting structural recurrence (Supplementary Data 5).

### Evaluation and comparison of the models

We evaluated the predictive performance of each model in the test dataset (Fig. 1B). First, we evaluated the discrimination of the models by using the area under the receiver operating characteristic (AUC-ROC) curve [12]. We also compared the AUC values of the ML models and the AUC values of the ATA risk stratification by using the DeLong test [36], and a 2-tailed test with *P* < 0.05 was considered to indicate statistical significance. We used the Youden index as the threshold to calculate the sensitivity, specificity, accuracy, positive predictive value (PPV), negative predictive value (NPV), and F1 score [37]. Second, we evaluated the calibration of the models by using calibration curves, which represent the accuracy of the absolute recurrent risk estimates of the models [12]. Finally, we analyzed the interpretability of the ML models by using the rank of variable importance. Statistical analyses were performed using python (version 3.6.10, https://www.python.org) and R software (version 4.2.2, https://www.r-project.org/).

## Results

### Characteristics of the study population

The median age of the 2244 patients was 42.0 years, and 1489 (66.4%) patients were female. During a median follow-up of 45.5 months (range: 12.0 to 142.7 months), 179 patients (8.0%) experienced structural recurrence (Table 1). The median tumor diameters of ≤ 10 mm, 10 to 20 mm, 20 to 40 mm, and > 40 mm were documented in 1093 (48.7%), 777 (34.6%), 322 (14.3%), and 52 (2.3%) patients, respectively. Multifocality was observed in 577 (25.7%) patients. Extensive ETE was observed in 584 (26.0%) patients. The median non-stimulated Tg was 0.24 ng/mL with median TSH was 0.46 µIU/ml. A total of 1537 (68.5%) patients and 698 (31.1%) patients underwent central LN dissection and lateral LN dissection, respectively. A total of 1173 (52.3%) and 564 (25.1%) patients had N1a and N1b disease, respectively. The median LNR was 28.57%. A total of 328 (14.6%), 1334 (59.4%), and 582 (25.9%) patients were classified as low risk, intermediate risk, and high risk, respectively, according to the ATA risk stratification.

**Table 1:** Characteristics of the patients in this study

Characteristics		N (%) or median (Q1, Q3)		
		Patients without recurrent disease (*N* = 2065)	Patients with recurrent disease (179)	Overall (*N* = 2244)
Demographic characteristics and comorbidities				
Age, years		41.00 (33.00, 50.00)	44.00 (35.50, 52.00)	42.00 (33.00, 50.00)
	≤ 42.0	1127 (54.6)	83 (46.4)	1210 (53.9)
	> 42.0	938 (45.4)	96 (53.6)	1034 (46.1)
Sex	Male	677 (32.8)	78 (43.6)	755 (33.6)
	Female	1388 (67.2)	101 (56.4)	1489 (66.4)
Race	Han	1983 (96.0)	171 (95.5)	2154 (96.0)
	Others	82 (4.0)	8 (4.5)	90 (4.0)
Smoking	No	1787 (86.5)	147 (82.1)	1934 (86.2)
	Yes	278 (13.5)	32 (17.9)	310 (13.8)
Alcohol drinking	No	1702 (82.4)	139 (77.7)	1841 (82.0)
	Yes	363 (17.6)	40 (22.3)	403 (18.0)
Comorbidity of diabetes	No	2000 (96.9)	165 (92.2)	2165 (96.5)
	Yes	65 (3.1)	14 (7.8)	79 (3.5)
Comorbidity of hypertension	No	1424 (69.0)	96 (53.6)	1520 (67.7)
	Yes	641 (31.0)	83 (46.4)	724 (32.3)
Comorbidity of Hashimoto’s thyroiditis	No	1898 (91.9)	161 (89.9)	2059 (91.8)
	Yes	167 (8.1)	18 (10.1)	185 (8.2)
Tumor-related variables				
Histology	PTC	2029 (98.3)	178 (99.4)	2207 (98.4)
	FV-PTC	36 (1.7)	1 (0.6)	37 (1.6)
Tumor diameter, mm	≤ 10	1026 (49.7)	67 (37.4)	1093 (48.7)
	10 to 20	707 (34.2)	70 (39.1)	777 (34.6)
	20 to 40	289 (14.0)	33 (18.4)	322 (14.3)
	> 40	43 (2.1)	9 (5.0)	52 (2.3)
Tumor foci	Unifocality	1550 (75.1)	117 (65.4)	1667 (74.3)
	Multifocality	515 (24.9)	62 (34.6)	577 (25.7)
Tumor location	Isthmus	9 (0.4)	0 (0.0)	9 (0.4)
	Left	788 (38.2)	64 (35.8)	852 (38.0)
	Right	902 (43.7)	73 (40.8)	975 (43.4)
	Bilateral	366 (17.7)	42 (23.5)	408 (18.2)
ETE	No	1385 (67.1)	112 (62.6)	1497 (66.7)
	Minimal	151 (7.3)	12 (6.7)	163 (7.3)
	Extensive	529 (25.6)	55 (30.7)	584 (26.0)
BRAF mutation	Negative	256 (12.4)	24 (13.4)	280 (12.5)
	Positive	543 (26.3)	49 (27.4)	592 (26.4)
	Unknown	1266 (61.3)	106 (59.2)	1372 (61.1)
Tg, ng/mL		0.22 (0.08, 0.71)	0.99 (0.12, 4.14)	0.24 (0.09, 0.79)
	< 1.08	1715 (83.1)	90 (50.3)	1805 (80.4)
	≥ 1.08	350 (16.9)	89 (49.7)	439 (19.6)
LN-related variables				
LN dissection	No	9 (0.4)	0 (0.0)	9 (0.4)
	Central dissection	1443 (69.9)	94 (52.5)	1537 (68.5)
	Lateral dissection	613 (29.7)	85 (47.5)	698 (31.1)
Number of LN dissected		10 (5, 20)	13 (7, 30)	10 (5, 20)
	< 21	1579 (76.5)	108 (60.3)	1687 (75.2)
	≥ 21	486 (23.5)	71 (39.7)	557 (24.8)
ENE	No	1985 (96.1)	160 (89.4)	2145 (95.6)
	Yes	80 (3.9)	19 (10.6)	99 (4.4)
LNR, %		27.78 (8.69, 50.00)	35.71 (23.31, 55.05)	28.57 (9.63, 50.00)
	< 22.70	891 (43.1)	42 (23.5)	933 (41.6)
	≥ 22.70	1174 (56.9)	137 (76.5)	1311 (58.4)
N stage	N0	484 (23.4)	23 (12.8)	507 (22.6)
	N1a	1093 (52.9)	80 (44.7)	1173 (52.3)
	N1b	488 (23.6)	76 (42.5)	564 (25.1)
Metabolic and inflammatory markers				
BMI, kg/m^2^	< 18.5	120 (5.8)	6 (3.4)	126 (5.6)
	18.5 to 24.0	1141 (55.3)	91 (50.8)	1232 (54.9)
	24.0 to 28.0	637 (30.8)	55 (30.7)	692 (30.8)
	≥ 28.0	167 (8.1)	27 (15.1)	194 (8.6)
Triglyceride, mmol/L		1.70 (1.70, 2.58)	1.63 (1.14, 2.43)	1.70 (1.70, 2.57)
	< 2.30	1428 (69.2)	126 (70.4)	1554 (69.3)
	≥ 2.30	637 (30.8)	53 (29.6)	690 (30.7)
Cholesterol, mmol/L		6.12 (5.32, 6.97)	6.34 (5.47, 7.17)	6.14 (5.34, 6.97)
	< 6.20	1091 (52.8)	85 (47.5)	1176 (52.4)
	≥ 6.20	974 (47.2)	94 (52.5)	1068 (47.6)
LDL, mmol/L		3.61 (3.01, 4.28)	3.82 (3.07, 4.35)	3.62 (3.01, 4.28)
	< 4.10	1423 (68.9)	110 (61.5)	1533 (68.3)
	≥ 4.10	642 (31.1)	69 (38.5)	711 (31.7)
HDL, mmol/L		1.55 (1.27,1.87)	1.58 (1.30, 1.94)	1.55 (1.27, 1.88)
	< 1.00	164 (7.9)	13 (7.3)	177 (7.9)
	≥ 1.00	1901 (92.1)	166 (92.7)	2067 (92.1)
NLR		1.59 (1.25, 2.08)	1.60 (1.22, 2.06)	1.59 (1.25, 2.08)
	< 2.32	1741 (84.3)	146 (81.6)	1887 (84.1)
	≥ 2.32	324 (15.7)	33 (18.4)	357 (15.9)
PLR		88.66 (67.61, 113.45)	86.42 (67.98, 112.78)	88.41 (67.58, 113.43)
	< 116.18	1580 (76.5)	144 (80.4)	1724 (76.8)
	≥ 116.18	485 (23.5)	35 (19.6)	520 (23.2)
PNI		55.85 (52.85, 58.85)	55.75 (53.33, 58.95)	55.83 (52.85, 58.90)
	< 56.15	1076 (52.1)	96 (53.6)	1172 (52.2)
	≥ 56.15	989 (47.9)	83 (46.4)	1072 (47.8)
LMR		7.68 (6.00, 9.96)	7.65 (5.88, 9.80)	7.67 (6.00, 9.95)
	< 6.47	644 (31.2)	65 (36.3)	709 (31.6)
	≥ 6.47	1421 (68.8)	114 (63.7)	1535 (68.4)
ATA risk stratification				
	Low risk	314 (15.2)	14 (7.8)	328 (14.6)
	Intermediate risk	1230 (59.6)	104 (58.1)	1334 (59.4)
	High risk	521 (25.2)	61 (34.1)	582 (25.9)

Abbreviations: PTC, papillary thyroid cancer; FV-PTC, follicular variant of papillary thyroid carcinoma; ETE, extrathyroid extension; Tg, thyroglobulin; LN, lymph node; ENE, extranodal extension; LNR, lymph node metastasis ratio; BMI, body mass index; LDL, low-density lipoprotein; HDL, high-density lipoprotein; NLR, neutrophil–lymphocyte ratio; PLR, platelet–lymphocyte ratio; PNI, prognostic nutritional index; LMR, lymphocyte–monocyte ratio; ATA, American Thyroid Association

Patients with recurrent disease were more likely to be older, male, be smokers, be drinkers, have comorbidities of diabetes, hypertension and Hashimoto’s thyroiditis, have larger, multifocal and bilateral tumors, have ETE, have higher levels of Tg, have undergone lateral dissection with a higher number of LN dissected and LNR, have ENE and more advanced N stage, have higher levels of BMI, cholesterol, LDL and NLR, and have lower levels of triglyceride, PLR, LMR and PNI.

### Performance of the models

The heatmap of the Spearman correlation analysis showed no significant or weak correlation between the majority of the input variables (Supplementary Data 6). The LASSO method selected nine variables for developing prediction models (Supplementary Data 7), including Tg, LN variables (LN dissection, number of LNs dissected, LNR, and N stage), comorbidities and metabolic-related variables (comorbidity of hypertension, comorbidity of diabetes, BMI, and LDL).

**Fig. 2:**
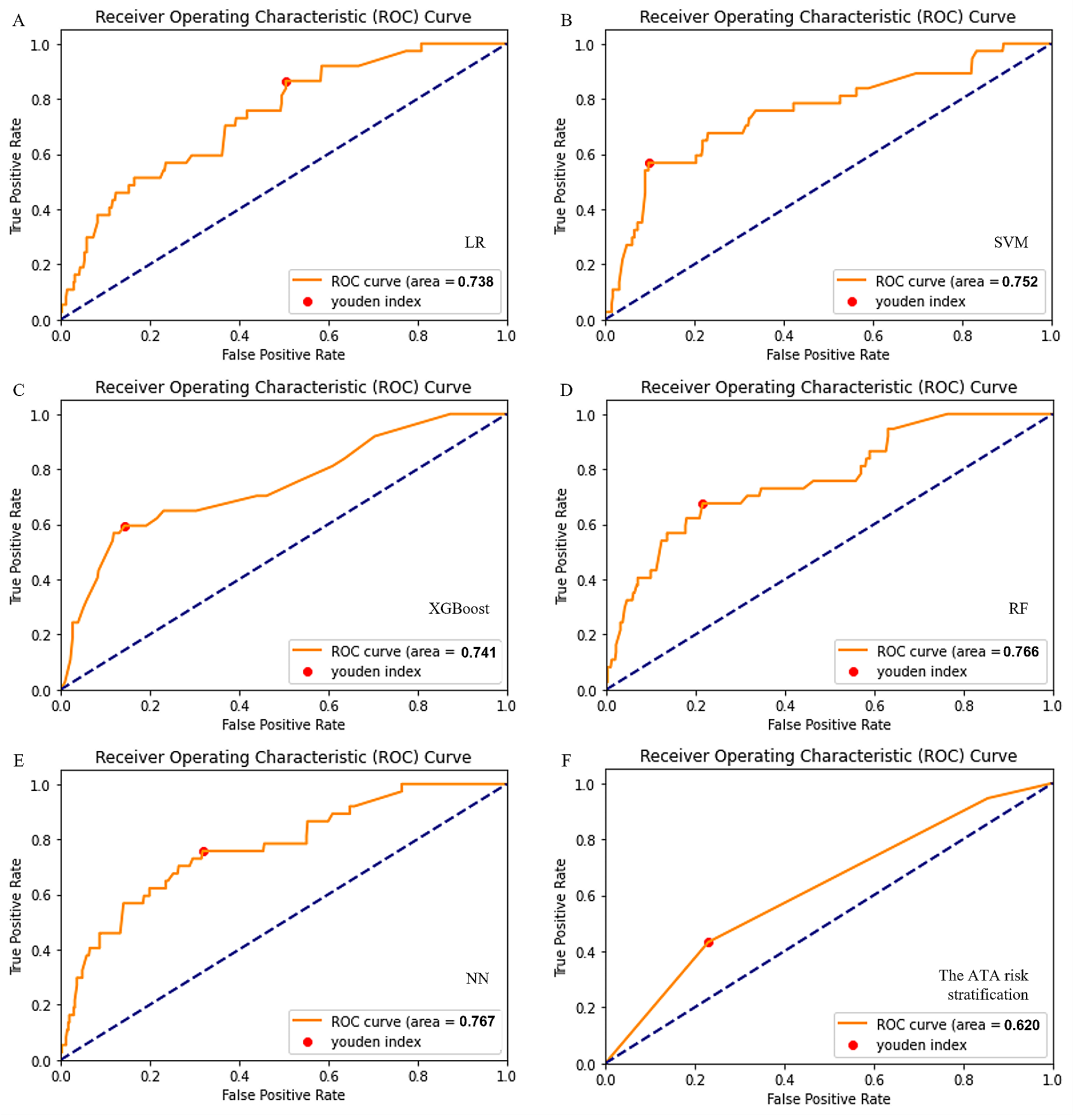
ROC curves of the models and the ATA risk stratification. LR, logistic regression; XGBoost, eXtreme gradient boosting; SVM, support vector machine; RF, random forest; NN, neural network; ATA, American Thyroid Association

**Fig. 3:**
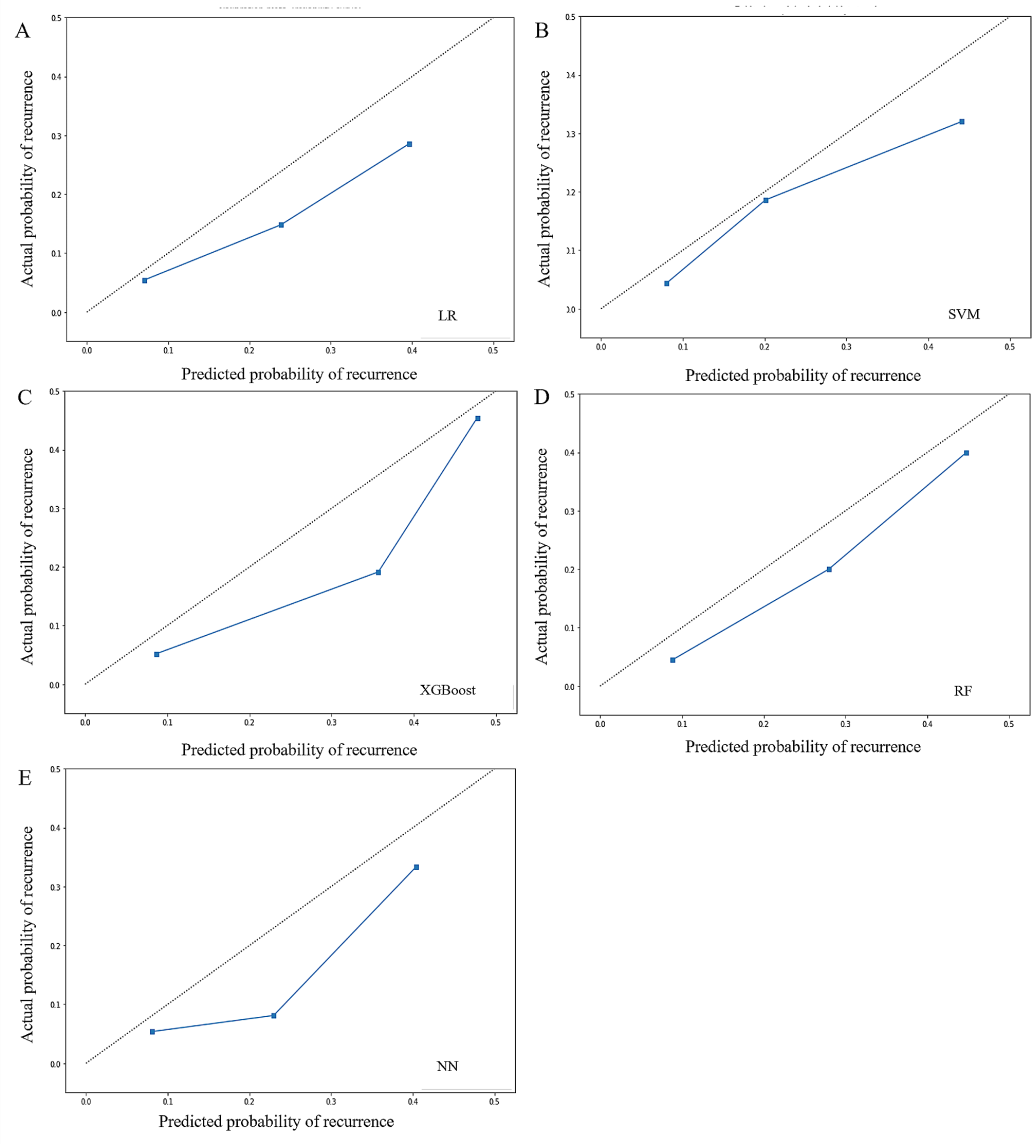
Calibration curves of the models. LR, logistic regression; XGBoost, eXtreme gradient boosting; SVM, support vector machine; RF, random forest; NN, neural network

As shown in Table 2; Fig. 2, five models had adequate discrimination in differentiating patients at greater risk of recurrence from those at lower risk, and the AUCs of the five models ranged from 0.738 to 0.767 in the test dataset (LR: AUC = 0.738, 95% CI = 0.636–0.820; SVM: AUC = 0.752, 95% CI = 0.666–0.841; XGBoost: AUC = 0.741, 95% CI = 0.609–0.840; RF: AUC = 0.766, 95% CI = 0.702–0.845; NN: AUC = 0.767, 95% CI = 0.675–0.843). All models showed better discrimination than did the ATA risk stratification (AUC = 0.620, 95% CI = 0.534–0.670; DeLong test: *P* < 0.01; Supplementary Data 8). The SVM, XGBoost, and RF model showed greater sensitivity (0.568, 0.595, 0.676), specificity (0.903, 0.857, 0.784), accuracy (0.875, 0.835, 0.775), positive predictive value (PPV) (0.344, 0.272, 0.219), negative predictive value (NPV) (0.959, 0.959, 0.964), and F1 score (0.429, 0.373, 0.331) than did the ATA risk stratification (sensitivity = 0.432, specificity = 0.770, accuracy = 0.742, PPV = 0.144, NPV = 0.938, F1 score = 0.216). The calibration curves are shown in Fig. 3. Although all models overestimated the recurrence risk of patients to varying degrees, which may have resulted in a higher false-positive rate if the models were applied in clinical practice, the RF model had generally consistent calibration.

**Table 2:** Predictive performance of the models in the test dataset

	AUC (95% CI)	Sensitivity	Specificity	Accuracy	PPV	NPV	F1 score
LR	0.738 (0.636–0.820)	0.865	0.495	0.526	0.133	0.976	0.231
SVM	0.752 (0.666–0.841)	0.568	0.903	0.875	0.344	0.959	0.429
XGBoost	0.741 (0.609–0.840)	0.595	0.857	0.835	0.272	0.959	0.373
RF	0.766 (0.702–0.845)	0.676	0.784	0.775	0.219	0.964	0.331
NN	0.767 (0.675–0.843)	0.757	0.682	0.688	0.176	0.969	0.286
The ATA risk stratification	0.620 (0.534–0.670)	0.432	0.770	0.742	0.144	0.938	0.216

Abbreviations: AUC, area under the curve; PPV, positive predictive value; NPV, negative predictive value; LR, logistic regression; SVM, support vector machine; XGBoost, eXtreme gradient boosting; RF, random forest; NN, neural network; ATA, American Thyroid Association

### Relative importance of variables in the models

**Fig. 4:**
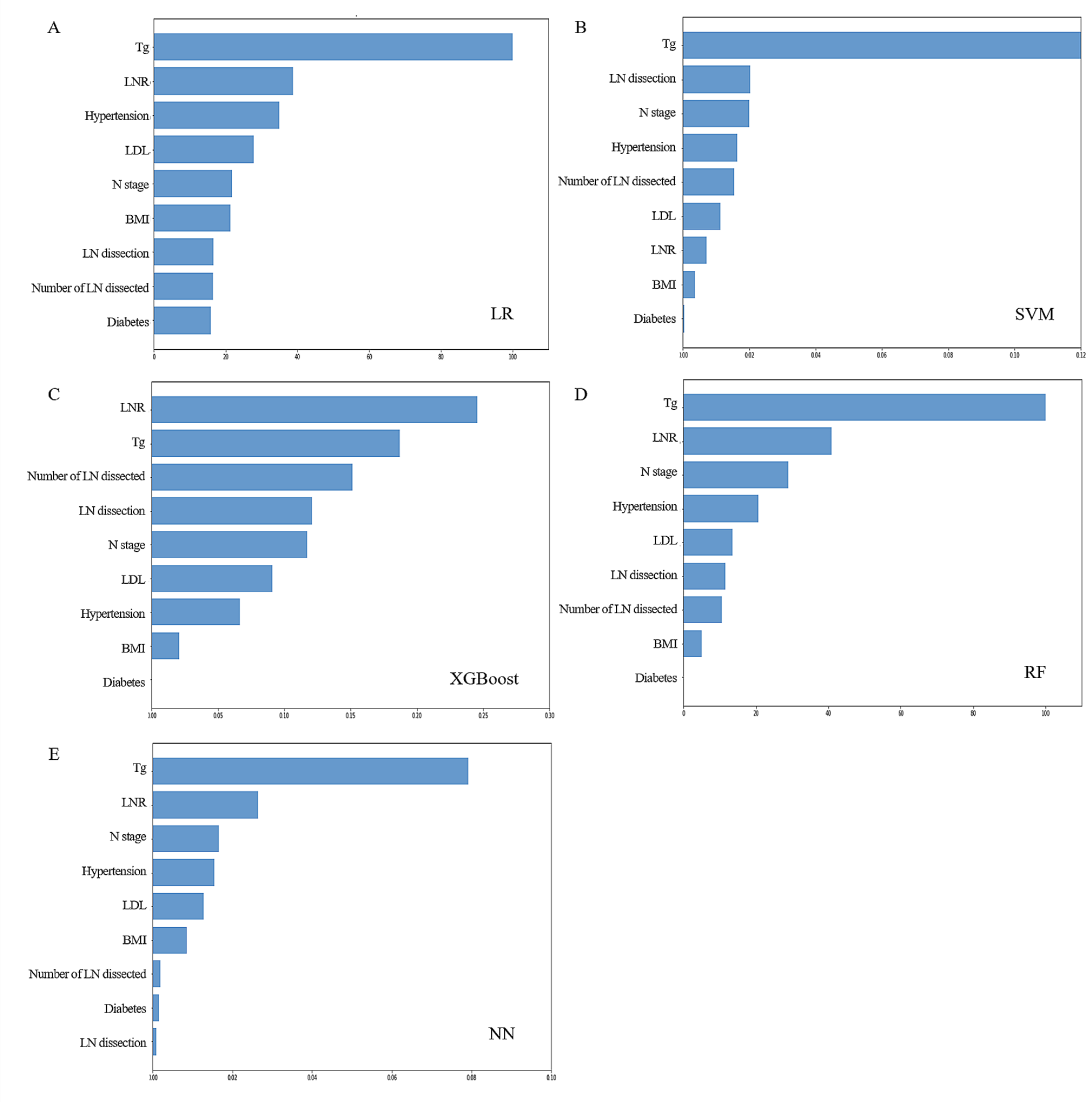
Relative importance of variables. LNR, lymph node metastasis ratio; Tg, thyroglobulin; LDL, low-density lipoprotein; BMI, body mass index; LR, logistic regression; XGBoost, eXtreme gradient boosting; SVM, support vector machine; RF, random forest; NN, neural network

Although slight differences were shown in the importance of variables among those models (Fig. 4), the Tg and the LNR were the top 2 important variables in all the models, the N stage was the top 5 important variables in all the models. The importance of variables in RF model was as follows: Tg, LNR, and N stage, comorbidity of hypertension, LDL, BMI, number of LNs dissected, comorbidity of diabetes, and LN dissection.

## Discussion

In this study, based on a large dataset of PTC patients with comprehensive predictive variables (demographic characteristics and comorbidities, tumor-related variables, LN-related variables, and metabolic and inflammatory markers), we developed and validated five ML models to predict structural recurrence in PTC patients. In the test dataset, the SVM, XGBoost, and RF model showed better discrimination than the ATA risk stratification according to the AUC values and corresponding indices, and the RF model generally had consistent calibration compared with the other models. Thus, ML models may aid in treatment decision making and improve postoperative prognosis for PTC patients by accurately estimating the likelihood of structural recurrence and identifying patients at high risk of recurrence. Overall, we suggested that the RF model, which showed overall good performance and interpretability, could be used to predict structural recurrence in patients with PTC.

Nine of 29 variables were selected by the LASSO method and used to develop models in this study, including the Tg, LN variables (LN dissection, number of LNs dissected, LNR, and N stage), comorbidities and metabolic markers (comorbidity of hypertension, comorbidity of diabetes, BMI, and LDL). Further variable importance analysis revealed that the Tg, LNR, and N stage were the three most important variables across all the models. Tg is the most important tumor marker in PTC, and the ATA risk stratification revealed abnormally elevated postoperative suppressed Tg as one of the high-risk predictors; however, it did not specify a cutoff value or include postoperative negative Tg in the postoperative recurrence risk assessment. In this study, a non-stimulated Tg value of 1.08 ng/mL was set as a cutoff by using the ROC curve, and patients with a higher level of non-stimulated Tg had a greater risk of recurrence than those with a lower level of Tg. Consistently, previous studies have used suppressed Tg values > 1 ng/mL to define a biochemical incomplete response to therapy in patients treated with total thyroidectomy and ^131^I ablation, and approximately 20% of these patients were likely to develop structural disease [3, 38].

LN-related variables were also considered important contributing predictors by the ML models. According to the 2015 ATA risk stratification system, the N stage and the size of the metastatic LN were proposed as key predictors for structural recurrence. In this study, we selected the number of dissected LNs, the LNR, and the N stage to develop models. The LNR was among the 2 most important variables, and the N stage was among the 5 most important variables according to all the models. A higher LNR (≥ 22.70%), greater number of LNs dissected (≥ 21), and advanced N stage were strongly associated with a high risk of recurrence. Several studies have reported various optimal cutoff values for LN-related variables [4–6]. For instance, in a recent study in which five ML models were constructed to predict recurrence among patients diagnosed with PTC, the LNR (cutoff = 0.24) and LN metastasis were identified as important variables [21]. Another study determined the predictive cutoff values for the number of metastatic LNs (4 and 13) and the LNR (0.28 and 0.58) using the K-means clustering algorithm [39]. The optimal predictive cutoff may depend on the extent of LN dissection, the number of LNs dissected, the annual number of surgeries performed by physicians, etc. Thus, more evidence is needed before combining LN-related variables with newly developed risk stratification or staging systems for PTC patients.

The comorbidities and metabolic-related markers (comorbidities of hypertension and diabetes, BMI, and LDL) were included in our models and showed potential predictive value. Although the current risk stratification or staging system for PTC does not include these predictors, a few studies have reported that hypertension [40], diabetes [41], a high level of BMI [11] and LDL [42] were significantly associated with the aggressiveness of PTC. The underlying mechanism between metabolism-related predictors and poor prognosis in PTC patients is less clear. Increasing insulin, insulin-like growth factor or TSH were associated with the aggressiveness of PTC in obese patients [43]. The LDL receptor played an important role in the RAS/RAF/MAPK (MEK)/ERK signaling cascade, and synergy between LDL-mediated receptor uptake and BRAF may lead to a worse prognosis in thyroid cancer patients [42].

Compared with existing studies on prognosis prediction for PTC [18–22], our study has several strengths. First, we developed ML models for predicting structural recurrence based on a large dataset of PTC patients. By using multiple ML algorithms, 5-fold cross-validation and Bayesian optimization, more reliable and robust predictions can be achieved. Second, benefitting from the simultaneous consideration of multiple predictors and the use of LASSO, we identified nine variables that were strongly associated with the risk of recurrence to develop models. Our results provided comprehensive evidence for the interpretation and clinical application of these predictors. Third, to help clinicians making optimal use of the models, we evaluated the discrimination, calibration, and interpretability of the ML models; however, these items were underreported in the published literature addressing PTC prognosis prediction [12, 44].

This study has several limitations. First, the retrospective nature of the study might have resulted in selection bias. Second, the ML models we developed were based on data from a single institution, and more studies covering wider populations are warranted for validation. Third, our models were built on data from patients diagnosed with PTC and treated with thyroidectomy and ^131^I; thus, they were unlikely to be accurate for patients whose tumor behavior was considerably different, such as children and adolescents with PTC [45] or patients who undergo lobectomy [46]. Fourth, our sample data used for developing the models were unbalanced due to the low incidence of recurrence in PTC patients (only 8.0% of patients experienced recurrence in our study). Unbalanced data may typically affect model training; thus, we performed resampling to minimize this effect. Advanced methods for handling imbalanced data have been proposed recently and need to be applied [47]. Fifth, 61.1% of the BRAF mutations were missing, which was used as a categorical variable and may affect the risk estimating of this variable. Finally, a median follow-up period of 45.5 months might be insufficient for the assessment of outcomes in PTC patients; thus, our models were mainly used to estimate short-term recurrence risk.

## Conclusion

This study demonstrated that the RF model achieved the expected prediction performance with generally good discrimination, calibration and interpretability. It is likely that ML approaches could improve the accuracy of the existing risk stratification for PTC as well as assist physicians in better understanding how ML approaches can be applied to optimize treatment and follow-up decisions.

### Electronic supplementary material

Below is the link to the electronic supplementary material.

Supplementary Material 1

## Data Availability

The datasets generated during and/or analyzed during the current study are available from the corresponding author upon reasonable request.

## Abbreviations

PTC: Papillary thyroid cancer
DTC: Differentiated thyroid cancer
ML: Machine learning
ATA: American Thyroid Association
^131^I: Radioiodine
BMI: Body mass index
ETE: Extrathyroid extension
ENE: Extranodal extension
LNR: Lymph node metastasis ratio
PTC: Papillary thyroid cancer
FV-PTC: Follicular variant of papillary thyroid carcinoma
Tg: Thyroglobulin
TSH: Thyrotropin
TgAb: Anti-thyroglobulin antibody
LDL: Low-density lipoprotein
HDL: High-density lipoprotein
NLR: Neutrophil–lymphocyte ratio
PLR: Platelet–lymphocyte ratio
LMR: Lymphocyte–monocyte ratio
PNI: Prognostic nutritional index
OSS: One-sided selection
LASSO: Least absolute shrinkage and selection operator
LR: Logistic regression
SVM: Support vector machine
XGBoost: EXtreme gradient boosting
RF: Random forest
NN: Neural network
BO: Bayesian optimization
AUC-ROC: The area under the receiver operating characteristic curve
PPV: Positive predictive value
NPV: Negative predictive value
