# Supplementary material for: Development and validation of prediction models for papillary thyroid cancer structural recurrence using machine learning approaches
Source: BMC Cancer. 2024 Apr 8;24:427. doi: 10.1186/s12885-024-12146-4 (PMC11000372; doi:10.1186/s12885-024-12146-4)
Supplement: Supplementary file 1 — Supplementary Material 1 [file 12885_2024_12146_MOESM1_ESM.docx]

**Supplementary Data**

**Contents**

[Supplementary Data 1. Missing value of 29 potential input variables and multiple imputations 2](#_Toc159848293)

[Supplementary Data 2. Details of 29 potential input variables in this study 3](#_Toc159848294)

[Supplementary Data 3. Details of the one-sided selection resampling 5](#_Toc159848295)

[Supplementary Data 4. Bayesian optimization process 5](#_Toc159848296)

[Supplementary Data 5. Methods of calculating the models’ variable importance 6](#_Toc159848297)

[Supplementary Data 6. Heat map of the Spearman correlation analysis 7](#_Toc159848298)

[Supplementary Data 7. Process of variable selection using the least absolute shrinkage and selection operator (LASSO) 8](#_Toc159848299)

[Supplementary Data 8. Comparison of the AUC-ROC of the models and the AUC-ROC of the ATA risk stratification by the DeLong test 8](#_Toc159848300)

# Supplementary Data 1. Missing value of 29 potential input variables and multiple imputations

In this study, the proportion of missing value of the Tg was 0.0%; the proportion of missing value of BRAF mutation was 61.1% which was then represented as a category (“Unknown”); the proportion of missing value of other 27 variables were <20% (range from 1.3% to 19.0%), so we used multiple imputations (MI) to handle these missing data as previous reported [1]. MI is an advanced method in handling missing values which is widely used in clinical research, and the distribution of imputated data is consistent with the original data. MI creates a number of datasets by imputing missing values, one missing value in original dataset is replaced by multiple plausible imputed values, these values take imputation uncertainty into consideration. Statistics of interest are estimated from each dataset and then combined into a final one. The R software (version 3.4.2) package ‘VIM’ (<https://www.rdocumentation.org/packages/VIM/versions/6.2.2>), and ‘mice’ (<https://www.rdocumentation.org/packages/mice/versions/3.16.0/topics/mice>) were used for MI.

# Supplementary Data 2. Details of 29 potential input variables in this study

| Potential input variables | | Details |
| --- | --- | --- |
| Demographic characteristics and comorbidities | | |
| Age, years | ≤42.0, >42.0 | Age at thyroid surgery.  The cut-off value was determined by using the ROC curve and Youden Index. |
| Sex | Male, Female | According to preoperative medical record |
| Race | Han, Others | According to preoperative medical record |
| Smoking status | No, Yes | According to preoperative medical record |
| Alcohol drinking status | No, Yes | According to preoperative diagnostic record |
| Comorbidity of diabetes | No, Yes | According to preoperative diagnostic record |
| Comorbidity of hypertension | No, Yes | According to preoperative diagnostic record |
| Comorbidity of Hashimoto's thyroiditis | No, Yes | According to preoperative diagnostic record and histopathological examination |
| Tumor-related variables | | |
| Histology | PTC, FV-PTC | According to histopathological examination |
| Tumor diameter, mm | ≥10, 10 to 20, 20 to 40, >40 | According to surgery record and histopathological examination |
| Tumor foci | Unifocality, Multifocality | According to surgery record and histopathological examination |
| Tumor location | Isthmus, Left, Right, Bilateral | According to surgery record and histopathological examination |
| ETE | No, Minimal, Extensive | According to surgery record and histopathological examination |
| BRAF mutation | Positive, Negative, Unknown | According to histopathological examination |
| Tg, ng/mL* | <1.08, ≥1.08 | Non-stimulated Tg (TSH<30 µIU/ml).  High-sensitive electrochemiluminescence immunoassay was used for the measurement of thyroglobulin (Elecsys® TG assay from 2006 to 2015; Roche Diagnostics, Mannheim, Germany, measuring range: 0.1-1000 ng/mL; Elecsys® TG II assay from 2016, Roche Diagnostics, measuring range: 0.04-5000 ng/mL).  The cut-off value was determined by using the ROC curve and Youden Index. |
| LN-related variables | | |
| LN dissection | No, Central dissection, Lateral dissection | According to surgery record |
| Number of LN dissected | <21, ≥21 | The cut-off value was determined by using the ROC curve and Youden Index |
| ENE | No, Yes | According to surgery record and histopathological examination |
| LNR, % | <22.70, ≥22.70 | The cut-off value was determined by using the ROC curve and Youden Index |
| N stage | N0, N1a, N1b | According to the 8^th^ AJCC TNM stage |
| Metabolic and inflammatory markers | | |
| BMI, kg/m^2^ | <18.5, 18.5 to 24.0, 24.0 to 28.0, ≥28.0 | BMI at thyroid surgery, obtained using the following formula: weight (kg) / height (m)^2^. The cut-off value was determined according to the Chinese BMI classification [2]. |
| Triglyceride, mmol/L* | <2.30, ≥2.30 | The cut-off value was determined according to the ACC/AHA guideline and ESC/EAS guidelines [3, 4] |
| Cholesterol, mmol/L* | <6.20, ≥6.20 | The cut-off value was determined according to the ACC/AHA guideline and ESC/EAS guidelines [3, 4] |
| LDL, mmol/L* | <4.10, ≥4.10 | The cut-off value was determined according to the ACC/AHA guideline and ESC/EAS guidelines [3, 4] |
| HDL, mmol/L* | <1.00, ≥1.00 | The cut-off value was determined according to the ACC/AHA guideline and ESC/EAS guidelines [3, 4] |
| NLR* | <2.32, ≥2.32 | Represented by the neutrophil/lymphocyte ratio.  The cut-off value was determined by using the ROC curve and Youden Index. |
| PLR* | <116.18, ≥116.18 | Represented by platelet/lymphocyte ratio.  The cut-off value was determined by using the ROC curve and Youden Index. |
| LMR* | <6.47, ≥6.47 | Represented by lymphocyte/monocyte ratio. The cut-off value was determined by using the ROC curve and Youden Index. |
| PNI* | <56.15, ≥56.15 | Obtained using the following formula: [albumin (g/L) + (5*lymphocyte count (10^9^/L)].  The cut-off value was determined by using the ROC curve and Youden Index. |

BMI, body mass index; PTC, papillary thyroid cancer; FV-PTC, follicular variant of papillary thyroid carcinoma; ETE, external thyroid invasion; LN, lymph node; ENE, extranodal extension; LNR, lymph node metastasis ratio; Tg, thyroglobulin; LDL, low-density lipoprotein; HDL, high-density lipoprotein; ACC, the American College of Cardiology; AHA, the American Heart Association; ESC, the European Society of Cardiology, EAS, the European Atherosclerosis Society. NLR, neutrophil lymphocyte ratio; PLR, platelet lymphocyte ratio; LMR, lymphocyte monocyte ratio; PNI, prognostic nutritional index

^*^Results within 1 to 6 months after the initial surgery and before radioiodine ablation, if there were more than one result, select the one closest to the date of surgery.

# Supplementary Data 3. Details of the one-sided selection resampling

The one-sided selection (OSS) resampling method was used to establish balanced training datasets. The OSS aims to remove noisy and borderline majority instances by adopting the concept of Tomek links. The technique works by randomly selecting a majority sample and combining it with all minority instances to create a newset. The newset is used to classify the original dataset and all misclassified instances are added to the newset. It considers majority instances to be redundant if these instances in newset participate in Tomek links [5]. The imblearn (version 0.12.0) was used for resampling.

# Supplementary Data 4. Bayesian optimization process


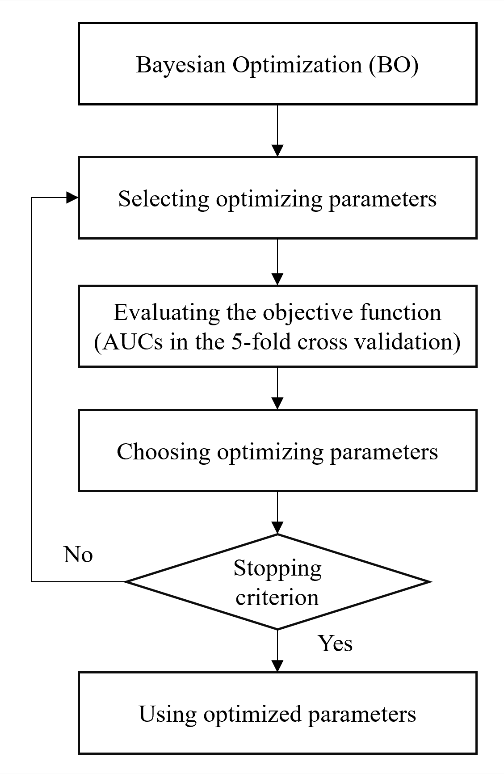


In this study, the hyperparameters for five machine learning models were optimized using Bayesian optimization (BO). Hyperparameters are a group of factors used in testing and training to support the learning process. The hyperparameters are stable throughout the training process, which improves model accuracy while simultaneously reducing memory usage and training time. Different models use different hyperparameters based on the problem description. BO is a hyperparameter tuning method for improving the accuracy of machine learning models. It seeks to collect observations that disclose as much information as possible about the function and the position of its optimal value with BO, and the ideal value might be discovered using relatively few samples. This approach does not require an explicit formulation of the function, in contrast to conventional optimization techniques [6]. The sklearn (version 1.4.1) was used for model developing and validation. The hyperopt was used for the Bayesian optimization.

# Supplementary Data 5. Methods of calculating the models’ variable importance

Relative importance of the LR model was based on the partial chi-square statistic minus the predicted degrees of freedom [7]. Relative importance of the SVM, the RF and the NN was based on the Mean Decrease Accuracy (MDA) [8]. Relative importance of the XGBoost was based on the Gain of variables, namely, the relative contribution of the corresponding variable to the model calculated by taking each variable's contribution for each tree in the model.

# Supplementary Data 6. Heat map of the Spearman correlation analysis


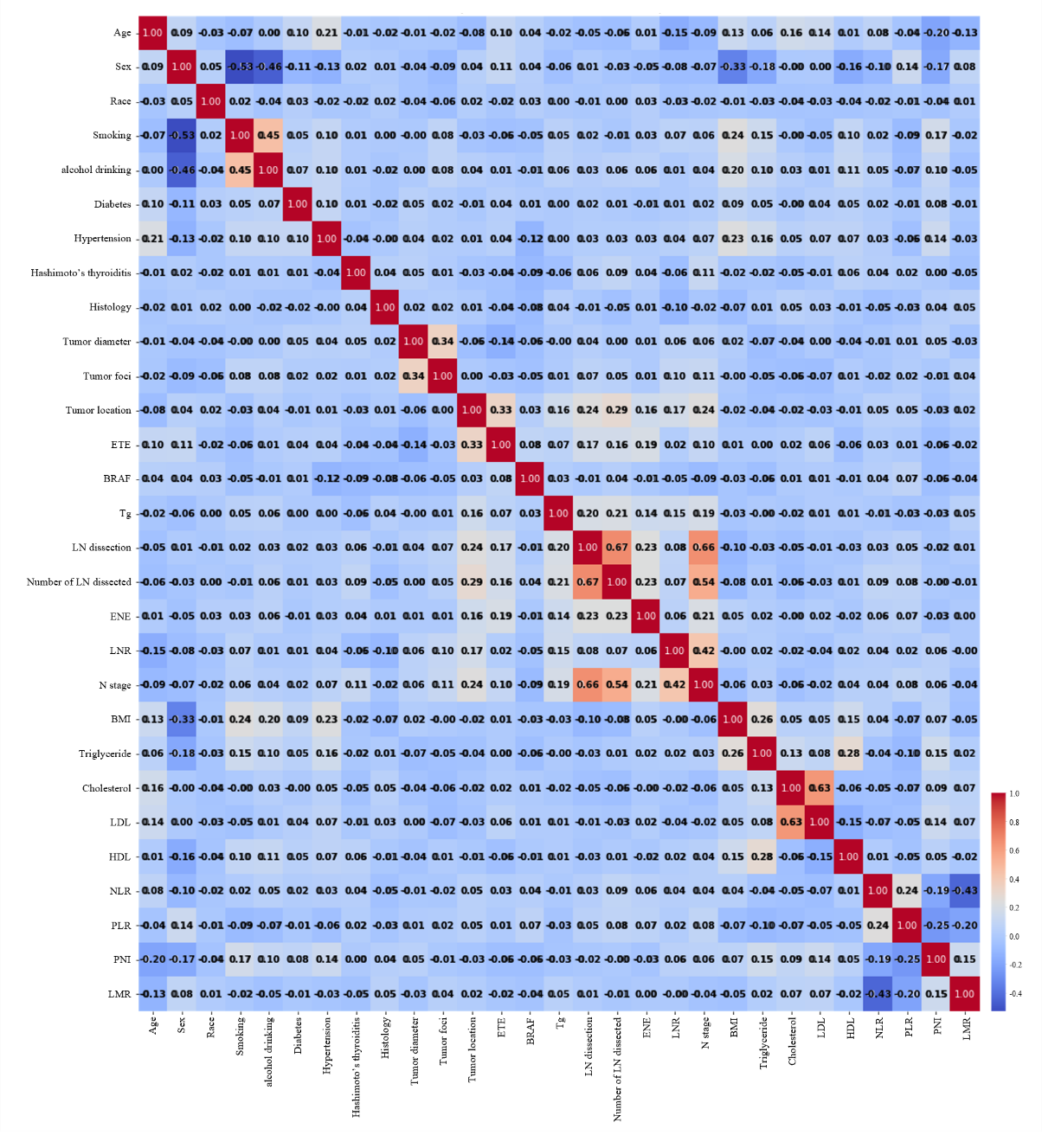


ETE, external thyroid invasion; Tg, thyroglobulin; LN, lymph node; ENE, extranodal extension; LNR, lymph node metastasis ratio; BMI, body mass index; LDL, low-density lipoprotein; HDL, high-density lipoprotein; NLR, neutrophil-lymphocyte ratio; PLR, platelet-lymphocyte ratio; LMR, lymphocyte-monocyte ratio; PNI, prognostic nutritional index

# Supplementary Data 7. Process of variable selection using the least absolute shrinkage and selection operator (LASSO)


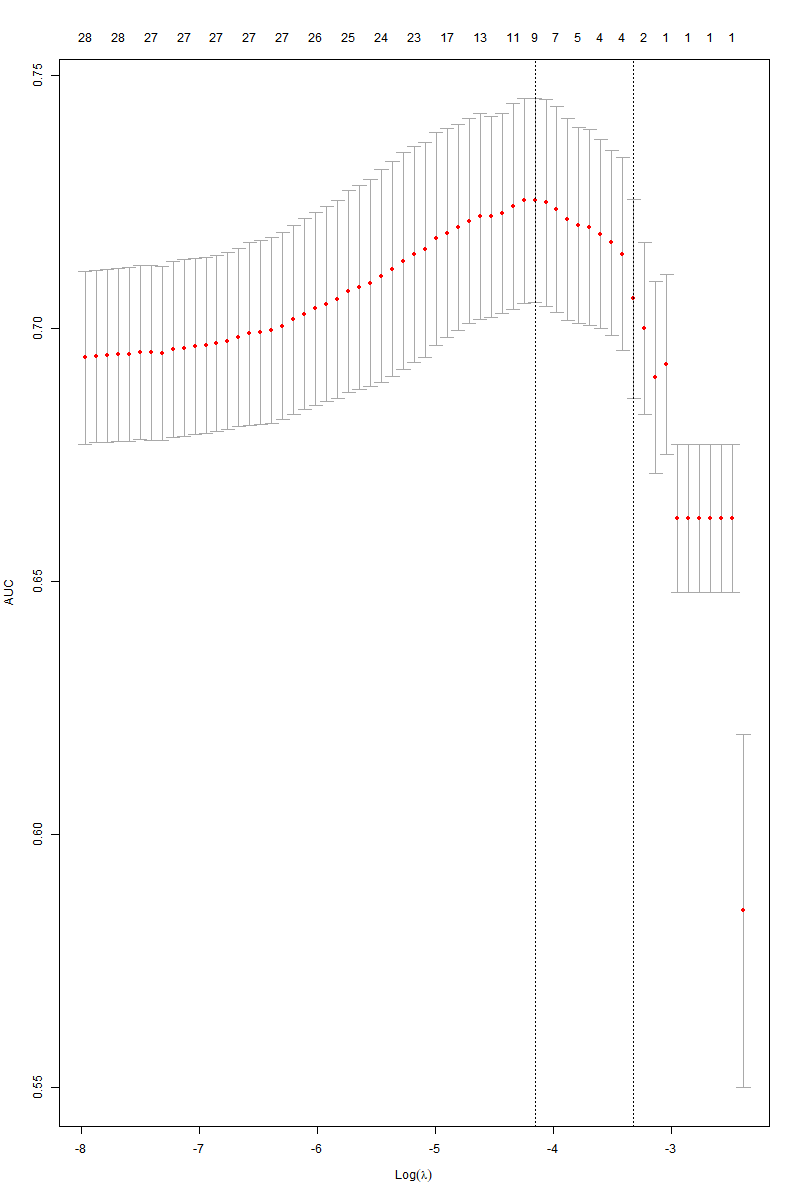


Variable selection for the prediction using the LASSO method, tuning parameter (λ) selection in the LASSO model involved the use of 5-fold cross validation with maximum AUC value.

# Supplementary Data 8. Comparison of the AUC-ROC of the models and the AUC-ROC of the ATA risk stratification by the DeLong test

|  | Z statistics | P value |
| --- | --- | --- |
| LR | 5.753 | <0.01 |
| SVM | 3.788 | <0.01 |
| XGBoost | 3.478 | <0.01 |
| RF | 5.346 | <0.01 |
| NN | 5.799 | <0.01 |

LR, logistic regression; XGBoost, eXtreme gradient boosting; SVM, support vector machine; RF, random forest; NN, neural network

**References**

1. Gravesteijn BY, Sewalt CA, Venema E, Nieboer D, Steyerberg EW: Missing Data in Prediction Research: A Five-Step Approach for Multiple Imputation, Illustrated in the CENTER-TBI Study. *J NEUROTRAUM* 2021, 38(13):1842-1857.

2. Pan XF, Wang L, Pan A: Epidemiology and determinants of obesity in China. *LANCET DIABETES ENDO* 2021, 9(6):373-392.

3. Stone NJ, Robinson JG, Lichtenstein AH, Bairey MC, Blum CB, Eckel RH, Goldberg AC, Gordon D, Levy D, Lloyd-Jones DM *et al*: 2013 ACC/AHA guideline on the treatment of blood cholesterol to reduce atherosclerotic cardiovascular risk in adults: a report of the American College of Cardiology/American Heart Association Task Force on Practice Guidelines. *J AM COLL CARDIOL* 2014, 63(25 Pt B):2889-2934.

4. Reiner Z, Catapano AL, De Backer G, Graham I, Taskinen MR, Wiklund O, Agewall S, Alegria E, Chapman MJ, Durrington P *et al*: ESC/EAS Guidelines for the management of dyslipidaemias: the Task Force for the management of dyslipidaemias of the European Society of Cardiology (ESC) and the European Atherosclerosis Society (EAS). *EUR HEART J* 2011, 32(14):1769-1818.

5. Bennin KE, Tahir A, MacDonell SG, B Rstler J: An empirical study on the effectiveness of data resampling approaches for cross-project software defect prediction. *IET SOFTW* 2022, 16(2):185-199.

6. C. H, B. Y, Y. L, X. Y: Automatic Parameter Tuning using Bayesian Optimization Method. In: *2019 IEEE Congress on Evolutionary Computation (CEC): 2019-1-1* 2019; 2019: 2090-2097.

7. Lyu Y, Liu Y, Xiao X, Yang Z, Ge Y, Jiang H: High level of intraoperative lactate might predict acute kidney injury in aortic arch surgery via minimally invasive approach in patients with type A dissection. *FRONT CARDIOVASC MED* 2023, 10:1188393.

8. Biau G, Scornet E: A random forest guided tour. *TEST-SPAIN* 2016, 25(2):197-227.
